# Supplementary material for: Duplications and Retrogenes Are Numerous and Widespread in Modern Canine Genomic Assemblies
Source: Genome Biol Evol. 2024 Jul 1;16(7):evae142. doi: 10.1093/gbe/evae142 (PMC11259980; doi:10.1093/gbe/evae142)
Supplement: evae142_Supplementary_Data [file evae142_supplementary_data.zip › Supplementary Figures_24_7_2.docx]

# Supplementary Figures


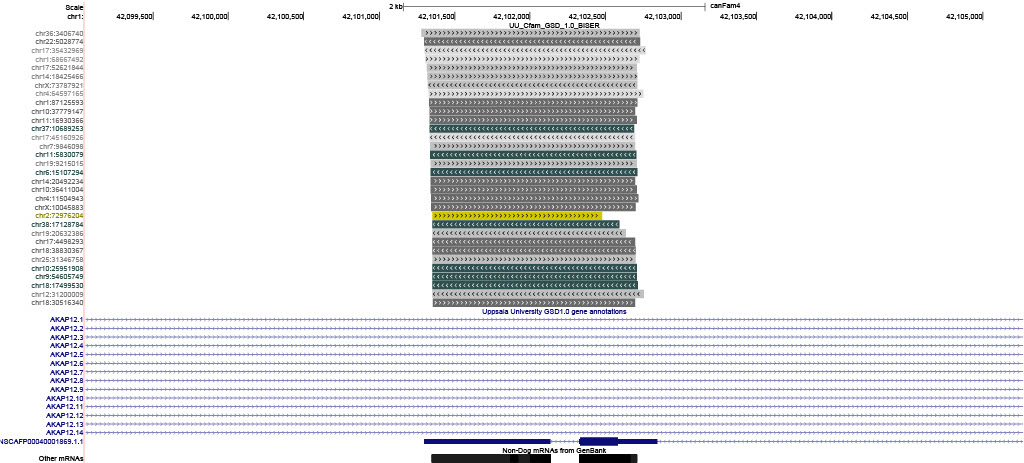


## Supplementary Figure 1. A High-Recurrence Duplicated Segment Detected by Genome Assembly Self-Alignment.

This screenshot of UCSC Genome Browser depicts the region chr1:42,099,050-42,105,269 in Mischka, a segment of the genome located in an intron of the gene *AKAP12*. The green and gray bars depict duplicated intervals detected by genome assembly self-alignment (BISER, top), whereas the blue lines with arrows indicate genes annotated in the Mischka assembly. The final track represents non-canine mRNAs from GenBank. Each gray/green bar has a location where the duplicated sequence is found elsewhere as indicated at locations given on the left at homology levels between 90 and 98%, with the singular yellow bar indicating an even greater homology between 98% and 99%. The parental retrogene for this duplicated sequence is *HMGN2*, a common parent gene.


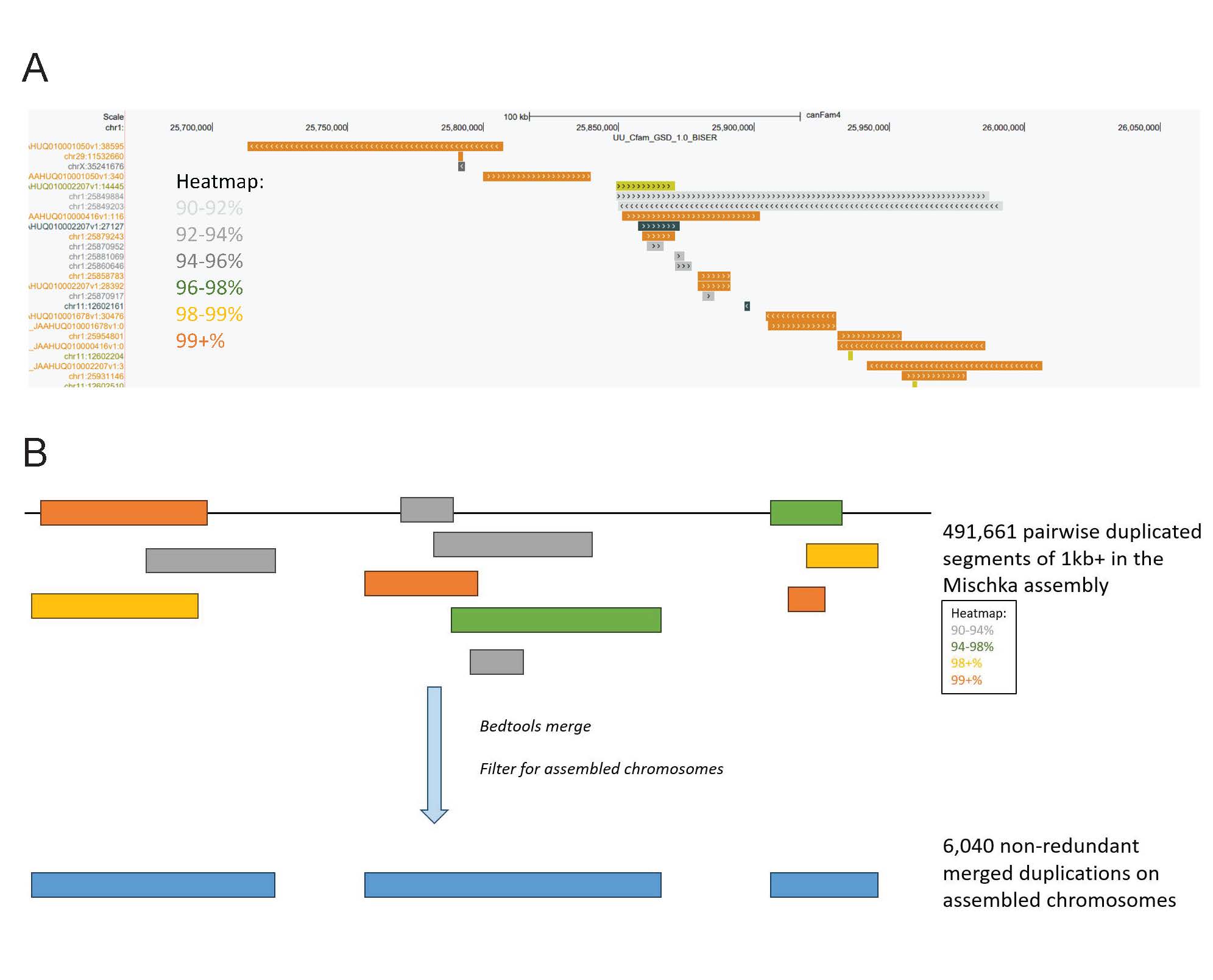


## Supplementary Figure 2. Merging Pairwise Duplications Identified by Genome Self Alignment.

In the genome assembly self-alignment analysis, we identify pairwise duplicated segments 1kb in size with greater than 90% sequence similarity (represented in color). These duplicated segments appear across all chromosomes present in canine assemblies, including on unplaced contigs. A) Because these duplications are pairwise, a single locus can be duplicated multiple times and be present in multiple pairwise alignments with varying levels of sequence similarity (depicted in color in UCSC Genome Browser). B) When merging, bedtools merge (v2.30) was used to combine overlapping segments together, regardless of sequence similarity We also removed duplications present on non-assembled chromosomes at this stage (chrM and unplaced contigs), since the treatment of unplaced contigs varies among the assemblies we studied.


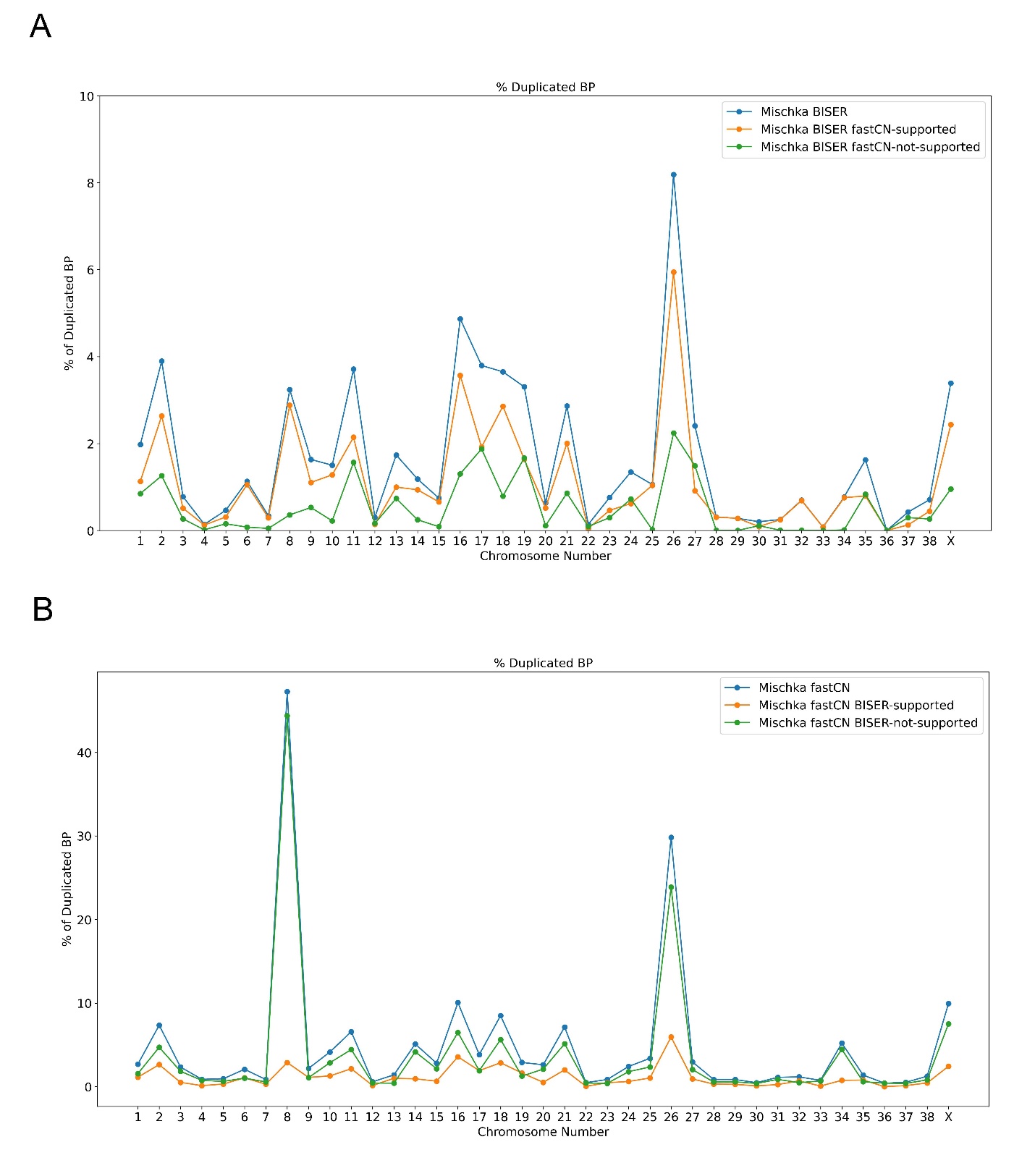


## Supplementary Figure 3. Duplication Content of Each Chromosome.

The X-axis represents each assembled chromosome, and the Y-axis represents what fraction of that chromosome is duplicated as a percentage. (A) Blue represents duplications found by genome assembly self-alignment (BISER). Orange is BISER duplications supported by read-depth, and green is unsupported. (B) Blue represents duplications detected by read-depth. Orange shows support from BISER, and green is unsupported.


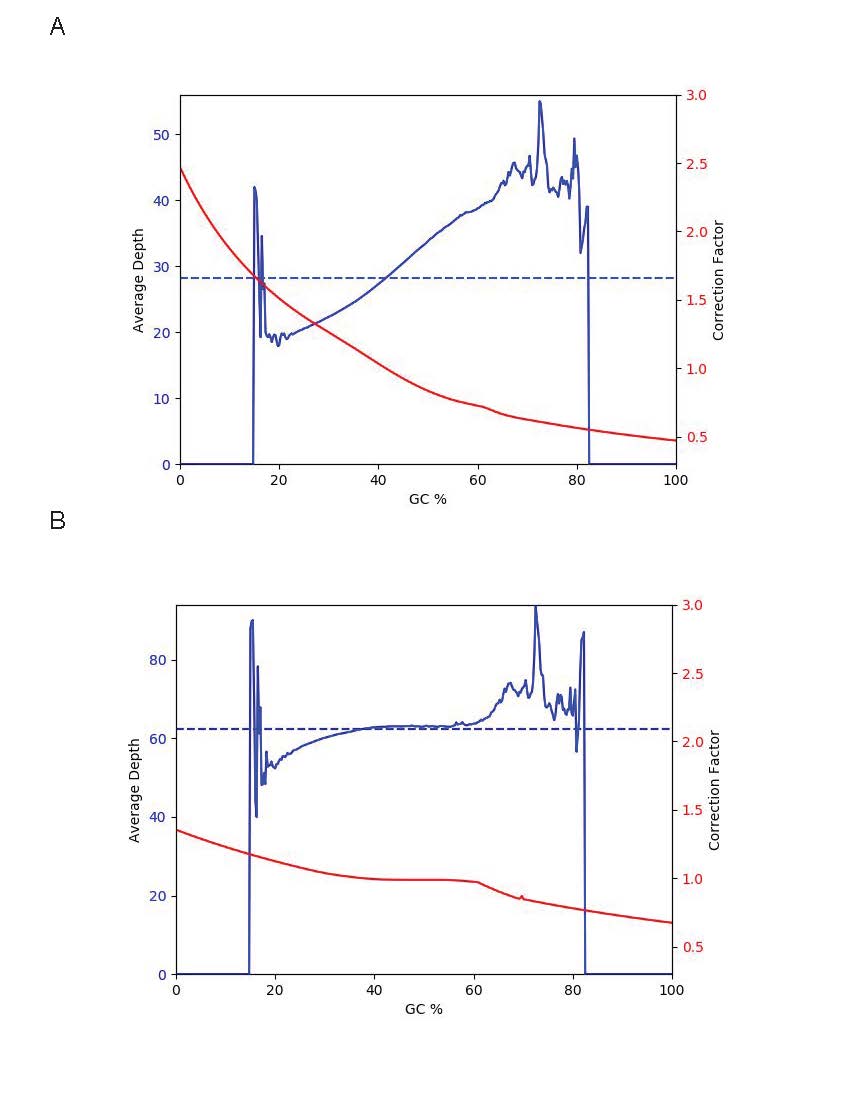


## Supplementary Figure 4. GC Coverage Profiles.

Plots of coverage by local GC content are produced by fastCN. The dashed blue line shows mean read-depth. The solid blue line shows the coverage as a function of local GC content. The red line shows the correction factor calculated to normalize coverage based on GC content. (A) Depicted is the curve for Nala, which shows a skewed coverage across different GC levels, suggesting bias in the Illumina data. (B) Depicted is the curve for Mischka, showcasing a more uniform coverage profile across GC levels.


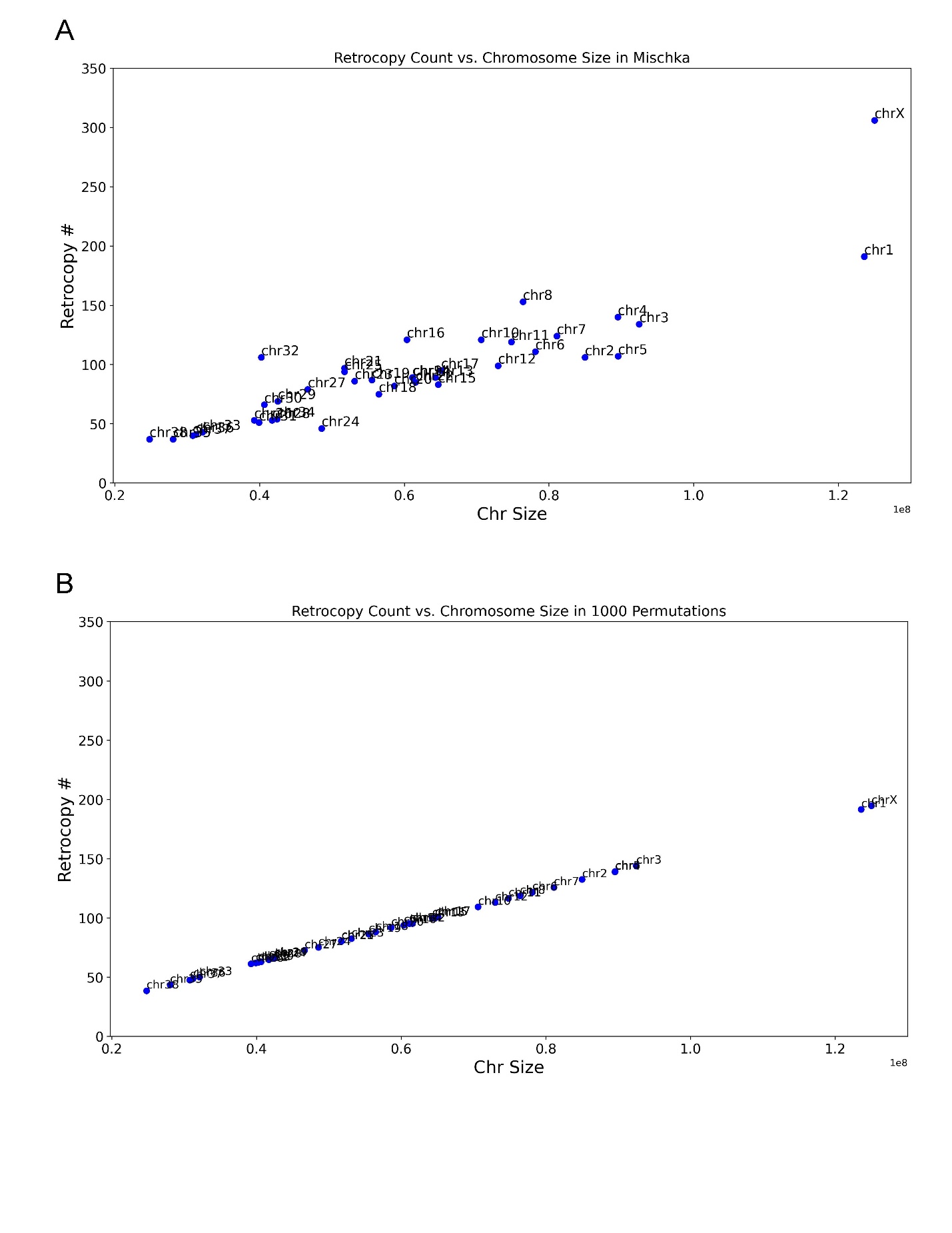


## Supplementary Figure 5. Retrocopy Count vs. Chromosome Size.

A) Depiction of the number of retrocopies on each of the assembled chromosomes (chr1-38 + X) found in the Mischka assembly vs. the length of each chromosome. ChrX has 228 total retrocopies. B) Depiction of the number of retrocopies on each of the assembled chromosomes found in 1000 random permutations vs. the length of each chromosome. ChrX has a mean of 146 retrocopies and a max of 188 retrocopies. Mischka contains a 1.18-fold increase of detected retrogenes on chrX.


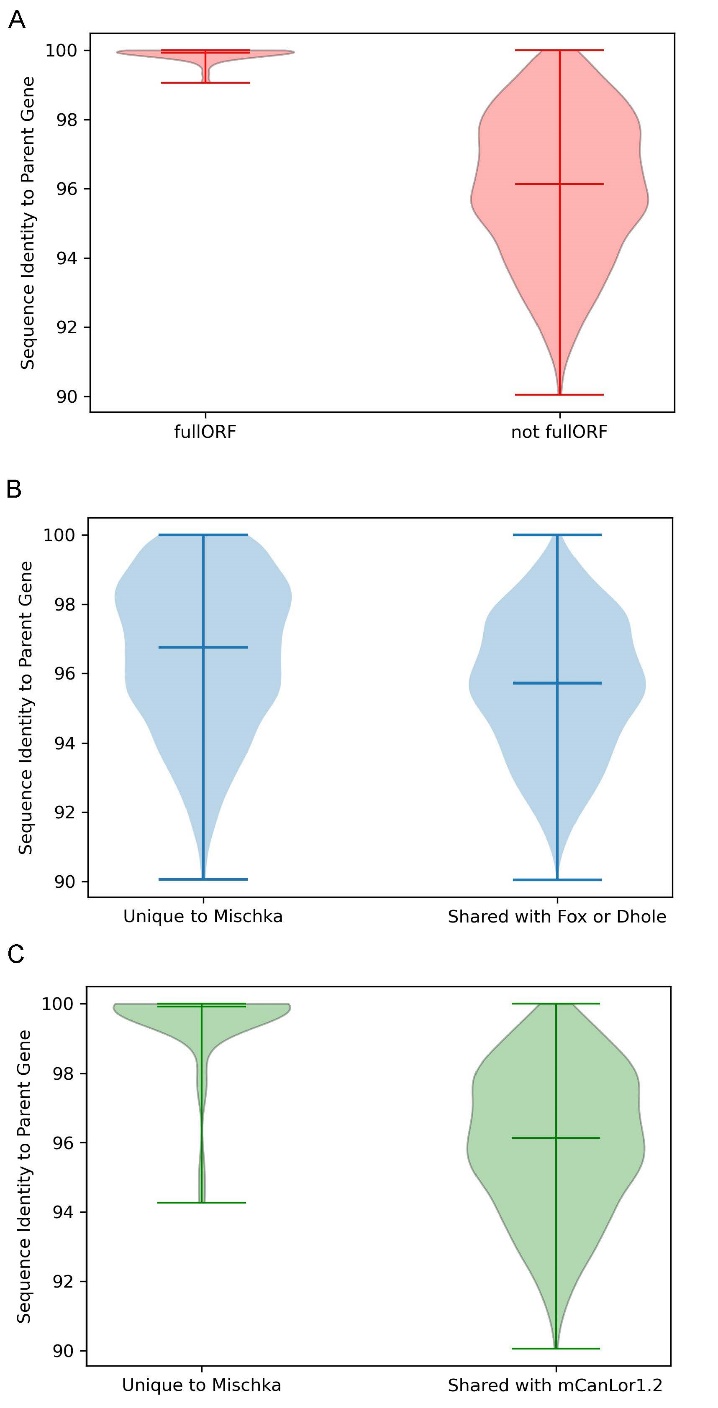


## Supplementary Figure 6. Sequence Similarity Between Mischka Retrocopies and Parent Gene

This violin plot depicts three categories of comparison of sequence similarity between retrocopies found in the Mischka assembly and the parent gene. A) Sequence similarity of retrocopies between those that retain the parental open-reading frame (Mean: 99.86%; Median: 99.93%) vs. those that do not (Mean: 96.04%; Median: 96.14%). P-value: 3.81*10^-48^. B) Sequence similarity of retrocopies unique to Mischka (Mean: 96.57%; Median: 96.75%) vs. those found in either the dhole or the fox assemblies (Mean: 95.62%; Median: 95.72%) P-value: 6.01*10^-44^. C) Sequence similarity of retrocopies unique to Mischka (Mean: 99.54%; Median: 99.92%) vs. those found in the mCanLor1.2 assembly (Mean: 96.04%; Median: 96.14%). P-value: 9.396*10^-29^.

##
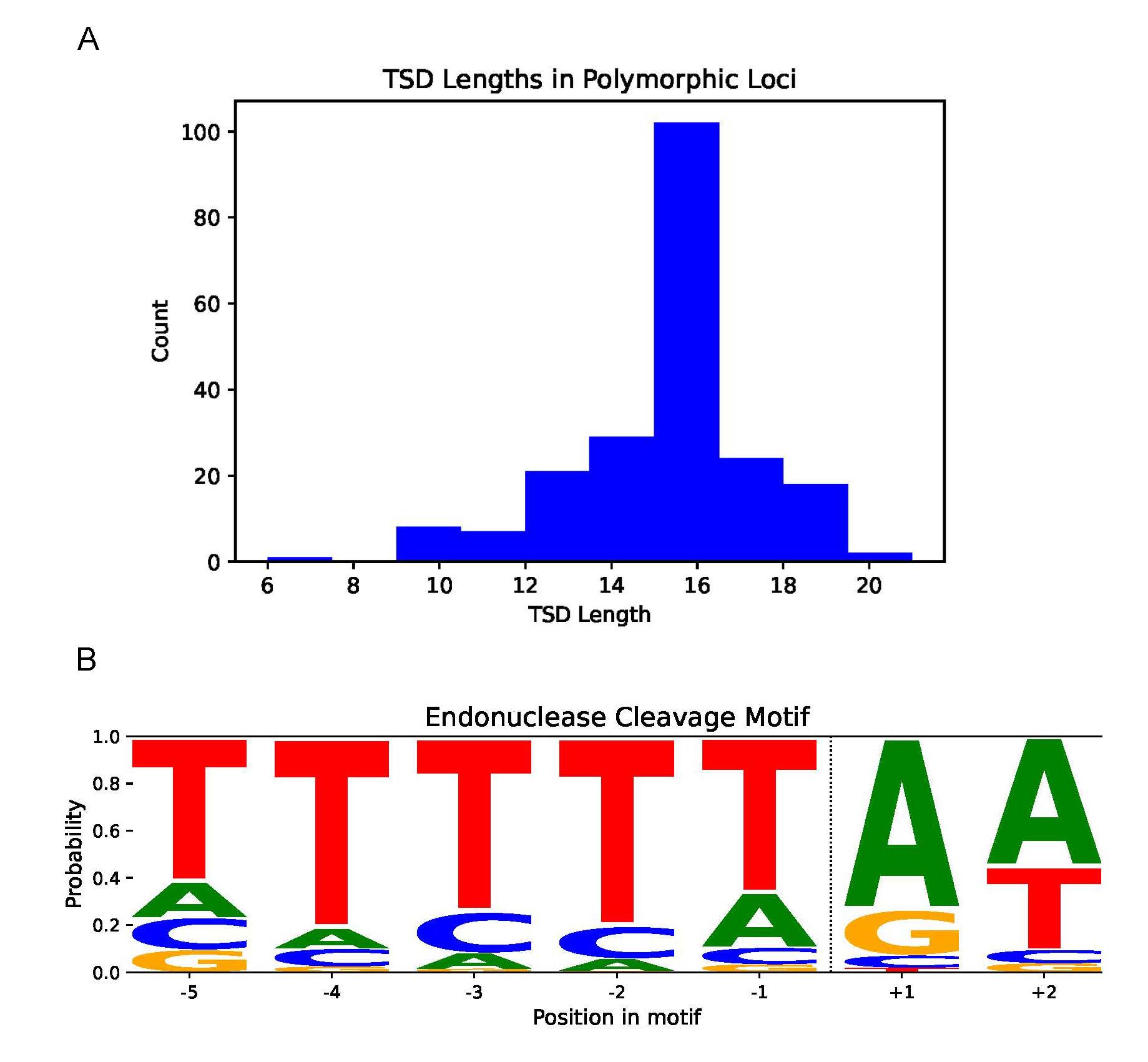
Supplementary Figure 7. Hallmark Analysis of Polymorphic Retrocopies.

An analysis of the polymorphic retrocopies (n=226 resolved loci). A) Detected TSD lengths for all polymorphic retrocopy containing loci with TSDs 5bp or longer (N=212). Only one canine is represented at each locus. Priority is given as to the first canine with an identified TSD as follows mCanLor, then Mischka, Sandy, Nala, China, and Zoey. The average TSD length across the dataset is 15.1 bp. B) The endonuclease cleavage motif for polymorphic loci containing 5bp TSDs, which follows the expected sequence of TTTTT/AA.


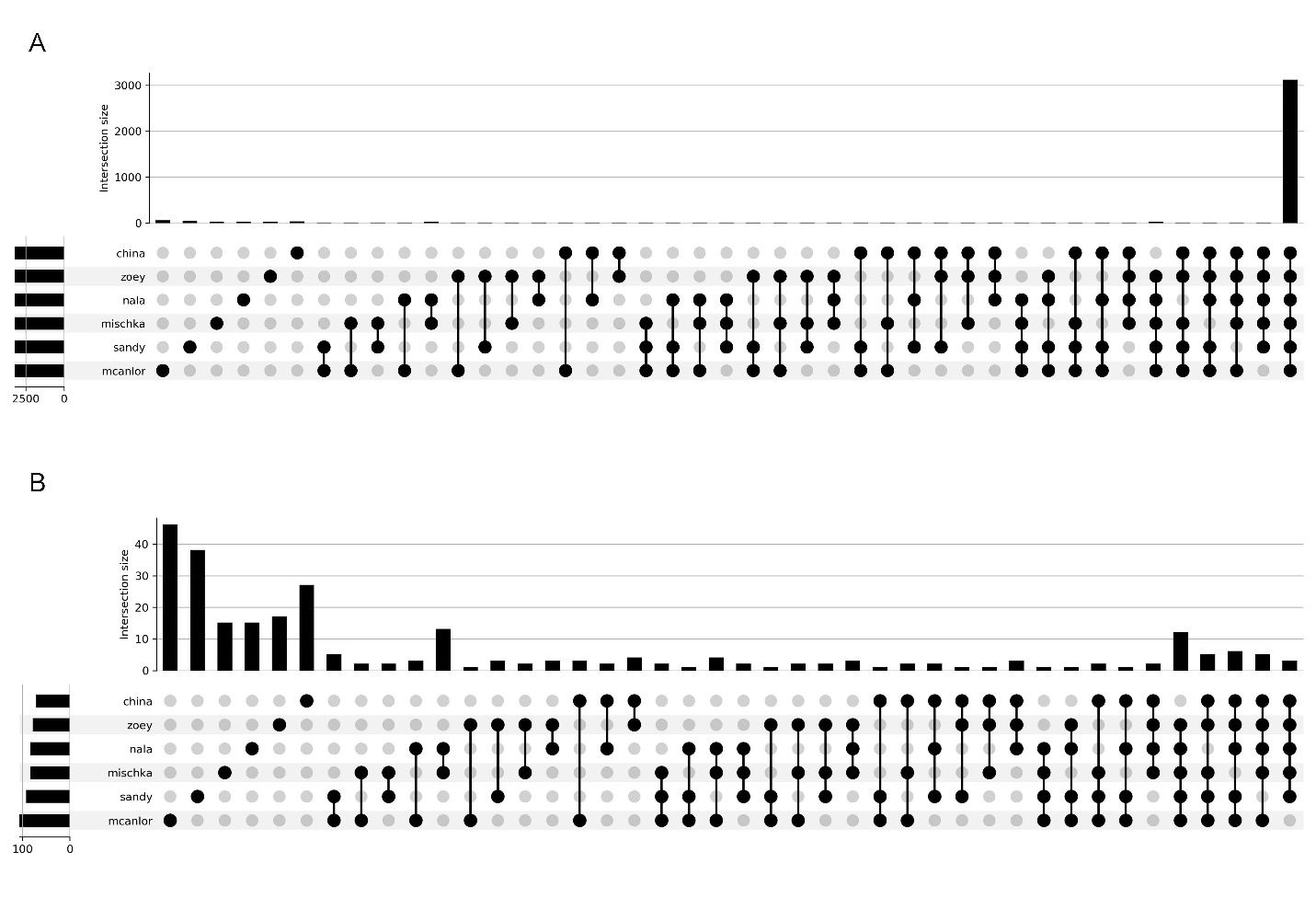


## Supplementary Figure 8. Retrocopies Shared Across Canine Assemblies.

This upset plot depicts 3,377 retrocopies that were found to be present in at least one assembly between Mischka, mCanLor1.2, Sandy, China, Nala, and Zoey. A) depicts all retrocopies grouped by presence in canine assemblies, and B) removes the category for shared between all six assemblies. Of the retrocopies depicted, 3,111 are shared between all six assemblies. The second largest category is mCanLor1.2-specific retrocopies (n=56). The remaining categories have between 1 and 38 retrocopies.


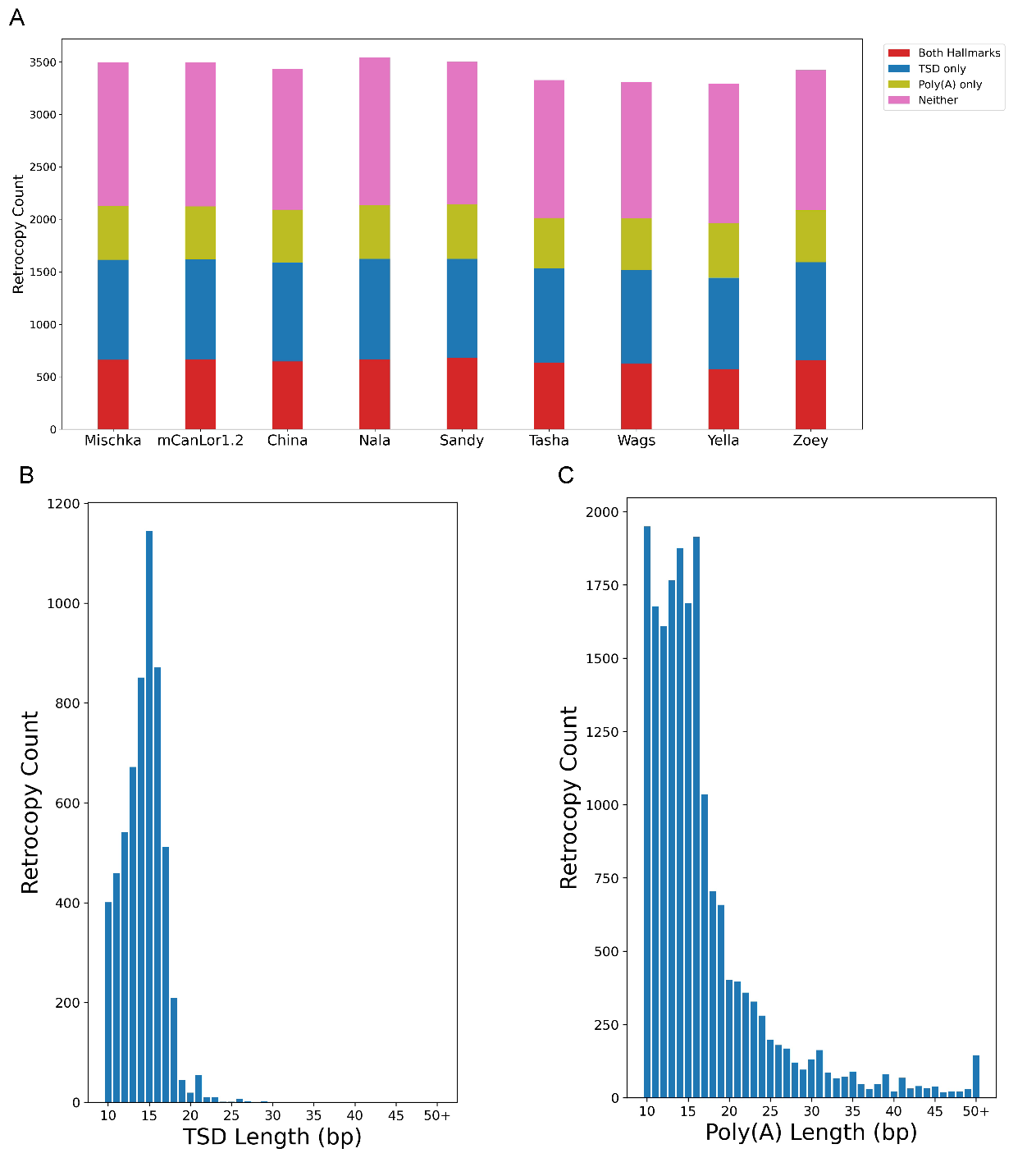


## Supplementary Figure 9. Retrocopy Hallmarks with Strict Calling.

These figures depict the retrocopy hallmarks in a similar fashion to Figure 3, but with the requirements of 10 bp TSDs, 10 bp poly(A)s, and that for a retrocopy to have both, the hallmarks must be within 5 bp of one another. A) The distribution of how many retrocopies possess TSDs, poly(A) tails, both, or neither in each of the nine canine assemblies. B) A histogram of the TSD lengths found for retrocopies in the Mischka assembly. TSD lengths of 50 or greater have been grouped into one bin. C) A histogram of the lengths of poly(A) tails found for retrocopies in the Mischka assembly. Lengths of 50 or greater have been grouped into one bin.
